# Why *Choo‐Choo* Is Better Than *Train*: The Role of Register‐Specific Words in Early Vocabulary Growth

**DOI:** 10.1111/cogs.12628

**Published:** 2018-07-11

**Authors:** Mitsuhiko Ota, Nicola Davies‐Jenkins, Barbora Skarabela

**Affiliations:** ^1^ School of Philosophy, Psychology and Language Sciences University of Edinburgh

**Keywords:** Infant‐directed speech, Baby‐talk words, Iconicity, Reduplication, Diminutives, Vocabulary development

## Abstract

Across languages, lexical items specific to infant‐directed speech (i.e., ‘baby‐talk words’) are characterized by a preponderance of onomatopoeia (or highly iconic words), diminutives, and reduplication. These lexical characteristics may help infants discover the referential nature of words, identify word referents, and segment fluent speech into words. If so, the amount of lexical input containing these properties should predict infants’ rate of vocabulary growth. To test this prediction, we tracked the vocabulary size in 47 English‐learning infants from 9 to 21 months and examined whether the patterns of growth can be related to measures of iconicity, diminutives, and reduplication in the lexical input at 9 months. Our analyses showed that both diminutives and reduplication in the input were associated with vocabulary growth, although measures of iconicity were not. These results are consistent with the hypothesis that phonological properties typical of lexical input in infant‐directed speech play a role in early vocabulary growth.

## 1 Introduction

In many languages and cultures, infant‐directed speech (IDS), or the speech to which infants and young children are exposed, systematically differs from adult‐directed speech (ADS) in several respects (see Saint‐Georges et al., 2013 and Soderstrom, 2007 for comprehensive reviews of the literature on the characteristics of IDS). Compared to ADS, IDS typically has slower speech rates, higher pitch ranges, and longer pauses (Cristia, 2013; Fernald, 1991, 1992). Lexical items in IDS are less diverse and more concrete (Phillips, 1973), and sentences in IDS tend to be shorter than those in ADS (Newport, Gleitman, & Gleitman, 1977). It has also been shown that at least some of the characteristics found in IDS are facilitative of language development. For example, infants’ performance is better in IDS than in ADS for tasks measuring categorization of phonetic segments (Trainor & Desjardins, 2002), segmentation of words (Thiessen, Hill, & Saffran, 2005), word–object associations (Ma, Golinkoff, Houston, & Hirsh‐Pasek, 2011), and detection of phrasal boundaries (Jusczyk et al., 1992).

These findings suggest that in some sense human infant learners are provided with a linguistic environment that is tailored for language learning. Most characteristics of IDS examined in the literature are, however, quantitative in nature. That is, the reported differences between IDS and ADS are matters of degree, for example, in speech rate, pitch range, pause length, lexical diversity, and sentence length, but not of elements or features that are unique to one register or the other. In fact, the same prosodic features typically found in IDS can also be elicited in ADS expressing heightened emotions (Trainor, Austin, & Desjardins, 2000). In this particular respect, IDS can be characterized simply as an exaggerated version of emotional ADS. Similarly, other characteristics of IDS, such as the reduced diversity in vocabulary and shorter sentences, do not amount to any qualitative modifications to the linguistic system either; the subset of ADS vocabulary and shorter sentences used in IDS are still part of the adult language in form and structure.

There is, however, a widely observed feature that is unique to IDS, and that is the set of lexical items that are specific to the register, such as *choo‐choo*,* tummy*, and *doggy* (Ferguson, 1964, 1977, 1978; Soderstrom, 2007). Known as ‘baby‐talk words’ or ‘infant‐directed vocabulary’, the majority of these words are conventionalized in the lexicon in the sense that they are established lexical items of the language rather than idiosyncratic words or expressions spontaneously produced by individuals. Yet the use of these words is highly contextualized to speech addressed to infants and children. Baby‐talk words can be found across languages of the world. Typological survey work shows that 25–60 such conventionalized register‐specific items can be identified in virtually every well‐documented language (Ferguson, 1964, 1978; Skarabela, Ota, Fazekas, & Wihlborg, 2015).

Whether baby‐talk words play any specific role in language development has been a matter of some debate (Falk, 2004; Ferguson, 1964, 1977; Laing, 2014; Locke, 2004; MacNeilage & Davis, 2004; Soderstrom, 2007). At first glance, baby‐talk words would seem to be an impediment to language learning. Introducing words that already have apparent lexical equivalents (e.g., *bunny* as well as *rabbit*;* tummy* as well as *stomach*) means adding redundancy to the lexicon and presenting misleading exceptions to mutual exclusivity. However, these costs may be counteracted by some benefits that these words bring into the context of learning. One such advantage has been addressed in research focusing on the phonetic characteristics of baby‐talk words, which are thought to be more suited to the developing articulatory skills of infants and young children. For example, MacNeilage and Davis (2004) report that baby‐talk words tend to contain consonant–vowel transitions that require the least amount of tongue movement (e.g., coronal consonant to front vowel; dorsal consonant to back vowel). Laing (2014) argues that some baby‐talk words, specifically onomatopoeia, bridge gaps in the production capability of young children because they permit ‘wild’ sounds that are not necessarily part of the phonemic inventory of the language but are nonetheless producible by infants. An analysis based on a metric of articulation difficulty also indicates that more iconic words (such as onomatopoeia) contain segments that are relatively easier to produce (Massaro & Perlman, 2017).

In the current study, we examine another way in which baby‐talk words may contribute to early language development. A growing body of literature is pointing to the possibility that baby‐talk words are more likely to be extracted and learned from the linguistic input than their adult lexical counterparts because they have certain characteristics that are in line with infants’ conceptual and perceptual predispositions. These effects are associated with three specific features that are frequently found in baby‐talk words: iconicity, diminutives, and reduplication. We hypothesize that these features bootstrap lexical learning by helping infants overcome the initial difficulties in understanding the nature of form‐meaning mappings in words, identifying the referents of word forms, and detecting word units in running speech. If this is the case, we predict that the initial advantages offered by words carrying these features can be leveraged to learn other words, thus further promoting lexical development. We elaborate on these ideas in the following sections, taking in turn each of the three typical characteristics of baby‐talk words mentioned above.

### 1.1 Iconicity

Many baby‐talk words exhibit highly iconic, or nonarbitrary, mappings between their form and meaning (Fernald & Morikawa, 1993; Imai & Kita, 2014). The canonical examples of this phenomenon in spoken language are onomatopoeic words depicting sounds produced by animals and vehicles, such as *baa‐baa*,* bow‐wow*,* quack*,* meow*,* choo‐choo*, and *vroom*. This tendency toward iconicity in IDS applies more generally to other words addressed to infants in both spoken and signed languages. For example, words used in English IDS, including those that are not specifically onomatopoeic, receive higher overall iconicity ratings than those in ADS (Perlman, Fusaroli, Fein, & Naigles, 2017; Perry, Perlman, Winter, Massaro, & Lupyan, 2017). In British Sign Language, signs with transparent iconicity are further modified in infant‐directed language in a way that highlights the nonarbitrary gesture‐meaning mapping (Perniss, Lu, Morgan, & Vigliocco, 2017).

There is now mounting experimental evidence that humans are biased to learn nonarbitrary sound‐meaning mappings (see Dingemanse, Blasi, Lupyan, Christiansen, & Monaghan, 2015 for a review of the relevant literature). For example, infants and toddlers are more likely to pay attention to or learn word‐object pairs with certain sound‐meaning correspondence, such as those exemplified by the nonsense words *kiki* and *bouba*, the former of which is perceived to be more congruent with a jagged object and the latter with a rounded object (Imai et al., 2015; Maurer, Pathman, & Mondloch, 2006; Ozturk, Krehm, & Vouloumanos, 2013). Infants also match the vowels [o] and [a] to large objects and [i] and [e] to small objects, demonstrating their sensitivity to vowel‐size symbolism (Peña, Mehler, & Nespor, 2011). Furthermore, toddlers are more likely to learn verb‐event pairs that are congruent with motion‐sound symbolism (Imai, Kita, Nagumo, & Okada, 2008; Kantartzis, Imai, & Kita, 2011). These effects are also found in naturalistic vocabulary development. Early‐acquired words tend to be more iconic than later‐acquired words both in spoken languages (Massaro & Perlman, 2017; Perlman et al., 2017; Perry, Perlman, & Lupyan, 2015; Perry et al., 2017) and sign languages (Caselli & Pyers, 2017; Thompson, Vinson, Woll, & Vigliocco, 2012).

These observations form the foundation of Imai and Kita's (2014) sound‐symbolism bootstrapping hypothesis, the notion that lexical iconicity promotes early word learning. Two specific claims associated with the hypothesis are directly relevant to our discussion of baby‐talk words. First, iconicity is facilitative because it affords learners with “the insight that speech sounds refer to entities in the world” (Imai & Kita, 2014: p. 4) — a prerequisite for lexical learning.^1^ This fundamental property of words is more accessible in words with transparent sound‐meaning mappings, such as onomatopoeic baby‐talk words. Second, iconicity may facilitate word learning because it alleviates Quine's problem: the challenge for learners to identify the exact meaning of a word when the context of word use allows for an unlimited number of possible interpretations. Nonarbitrary form‐meaning mappings offer one type of aid to this challenge by narrowing down the range of potential referents to those that have a good match with the phonological characteristics of the word. Thus, the baby‐talk word *woof‐woof* may signal to the learner that the word refers to the animal that is known to produce such vocalization, whether the connection between dogs and barking has been established by the learner's own observation or their parents’ rendition of a barking dog. The ideas behind the sound‐symbolism bootstrapping hypothesis suggests that highly iconic baby‐talk words are advantageous because they help young infants discover that words/signs associate sounds or gestures with meanings, and identify the specific sound‐meaning mappings in the face of referential indeterminacy.

### 1.2 Diminutives

Another feature that is frequently found in baby‐talk words is the use of diminutives, as exemplified by words such as *doggy*,* daddy*, and *tummy* (Berko Gleason, Perlmann, Ely, & Evans, 1994; Ferguson, 1964; Kempe, Brooks, & Gillis, 2007a). The use of diminutive affixes is extremely common in some languages such as Lithuanian, Russian and Spanish, in which as much as 40% of nouns used in IDS may be diminutivized (Kempe, Brooks, & Pirott, 2001; Savickiene, 1998). The productivity of diminutivization as a morphological operation is somewhat more constrained in other languages such as English, in which many apparent diminutive forms are lexically frozen (e.g., *tummy, kitty*, and *bunny*). But even in these languages, diminutives are used by the majority of caregivers with their infants (Berko Gleason et al., 1994).

Two aspects of diminutives have been linked to facilitation of lexical learning. First, diminutivization tends to regularize the prosodic shape of words (Echols, Crowhurst, & Childers, 1997; Jusczyk, 1997). In English, the majority of diminutives have a disyllabic structure with initial stress (e.g., *doggy, kitty, tummy, daddy*). In Spanish, diminutives move the location of stress to the canonical penultimate position (*teLEfono* > *telefoNIto* ‘telephone’). The second effect brought about by diminutives is that the affix makes the edges of words less variant (Kempe, Brooks, & Gillis, 2005; Kempe, Brooks, Gillis, & Samson, 2007b). In English, for example, words like *doggy*,* kitty*,* tummy*, and *daddy*, all end in the sound /i/, making the right‐edge of these words uniform. Both prosodic regularity and edge invariance are known to be important cues for word segmentation. Predominant rhythmic patterns in a language provide useful means to detect word boundaries (Cutler & Norris, 1988). In English, infants can use the predominant strong‐weak stress pattern of the language to segment words, making words like *doggy* and *kitty* likely units to be parsed as potential words in running speech (Jusczyk, Houston, & Newsome, 1999). Recurrent segmental patterns resulting from affixation also imply word boundaries and therefore can serve as cues for segmentation. Experiments with adults exposed to an unknown foreign language show that learners segment words from fluent speech better when their endings are made invariant through diminutivization (Kempe et al., 2005, 2007a,b), an effect that is likely extendable to infant learners.

### 1.3 Reduplication

The third property frequently found in baby‐talk words is reduplication, the full or partial repetition of syllables within a word (Ferguson, 1964, 1977; Gervain & Werker, 2008). English offers many examples of full reduplication such as *choo‐choo*,* night‐night*,* wee‐wee*, and *din‐din* as well as partial reduplication such as *daddy, tick‐tock*, and *bow‐wow*. The latter also includes compounded forms such as *teeny‐weeny* and *easy‐peasy*. The term *reduplication* in this context is used only as a description of the phonological structure of lexical items and it does not imply the type of repetition employed as a morphological operation in some (adult) languages to mark plurality, iterativity, intensity, or other morpho‐semantic features (e.g., Malay: *rumah* ‘house’ vs. *rumah*‐*rumah* ‘houses’).

Recent experimental work shows that reduplication can facilitate infants’ word segmentation and word learning. For example, 9‐month‐olds are better at segmenting reduplicated nonwords such as *neenee* and *foofoo* than similar but nonreduplicated nonwords such as *neefoo* and *foonee* from passages containing these words (Ota & Skarabela, 2018). Similarly, 18‐month‐olds are better at learning labels for unfamiliar objects when the labels are reduplicated (e.g., *neenee, foofoo*) than when they are not (e.g., *neefoo, foonee*) (Ota & Skarabela, 2016). These findings support the broader claim that human learners are constrained by perceptual or memory primitives that make them particularly sensitive to identity relations or repetitions of elements (Endress, Nespor, & Mehler, 2009). Furthermore, they are also consistent with findings that neonates show stronger neural responses to auditory sequences containing reduplicated syllables (e.g., *mubaba*) in comparison to sequences without repetition (e.g., *mubage*) (Gervain, Berent, & Werker, 2012; Gervain, Macagno, Cogoi, Peña, & Mehler, 2008). Thus, baby‐talk words with reduplication (e.g., *choo‐choo* and *night‐night*) may be more likely to be noticed in the input and stored in verbal memory than their adult‐like counterparts (e.g., *train* and *good night*), making them accessible targets for initial word learning.

### 1.4 The impact of baby‐talk words on global vocabulary development

The literature reviewed above suggests that typical features of baby‐talk words may facilitate early lexical development in several ways. Iconicity reveals the referential nature of words and provides sound‐meaning mappings that are more easily identifiable than others. Diminutivization aids word segmentation by making words comply with the canonical rhythmic patterns of the language and signaling the endings of words. Reduplicated forms may be more detectable in the input stream and also more easily committed to verbal memory. Indeed, across languages, the list of first words that young children comprehend or produce include many words characterized by iconicity, diminutives, or reduplication (e.g., English: *mommy*,* daddy*,* baa‐baa*,* woof*,* yum‐yum*; Italian: *mamma* ‘mommy’, *papa* ‘daddy’, *bau‐bau* ‘dog’, *pappa* ‘food/meal’, *nanna* ‘sleep’) (Caselli et al., 1995).

In this paper, we propose that the contribution of baby‐talk words to lexical development extends beyond the learning of individual words with iconicity, reduplication, and diminutive suffixes. As reviewed above, highly iconic words provide infants with referential insights into sound‐meaning mappings. Once infants discover this fundamental nature of words, they should be able to apply the same knowledge to other words. Similarly, if diminutives and reduplicated words ease segmentation and are easily learnable, then they can serve as lexical cues for further speech segmentation and learning. A similar idea has been demonstrated with early‐acquired words such as infants’ own name and appellations of parents (e.g., *mommy*/*mom*,* daddy*/*dad*), which 6‐month‐old infants can use to segment unknown adjacent words (Bortfeld, Morgan, Golinkoff, & Rathbun, 2005; Sandoval & Gómez, 2016). In other words, we hypothesize that baby‐talk words aid early lexical development not only because they themselves are easier to learn but also because they unveil the fundamental form‐meaning mapping nature of words and serve as anchor points for further word segmentation.

### 1.5 The current study

If the hypothesis that baby‐talk words can facilitate general lexical development is correct, then we expect individuals who receive more lexical input matching the characteristics of baby‐talk words to show some advantages in vocabulary growth. The purpose of this study was to test this prediction. Of course, from the viewpoint of the infant learner, it makes no difference whether the word they have heard happens to be unique to the register of IDS or not. We therefore make no distinction between baby‐talk words and non‐baby‐talk words in our analysis, but rather measure the overall proportions of words in the input that have properties corresponding to iconicity, diminutives and reduplication. The prediction is that infants who receive lexical input with a higher incidence of these features should have a faster overall rate of lexical growth at the initial stage of vocabulary development.

We tested this prediction on the overall vocabulary growth in longitudinal data from 47 English‐learning infants from the 9th to 21st month. We took measures of iconicity, diminutives and reduplication in the lexical input addressed to these infants at 9 months and examined their relationship to the infants’ rates of vocabulary size change for the following 1‐year period. We selected the age period from 9 to 21 months for two reasons. First, 9 months is the earliest age when individual differences in vocabulary estimates can be reliably obtained through currently available tools. Second, several longitudinal studies have found associations between infants’ speech segmentation skills between 7 and 12 months and their productive vocabulary size around 2 years (Newman, Bernstein Ratner, Jusczyk, Jusczyk, & Dow, 2006; Singh, Steven Reznick, & Xuehua, 2012). The one‐year period we selected roughly overlaps with the studies whose focus is related to one of the ways through which baby‐talk words are thought to contribute to general vocabulary development (i.e., the relationship between speech segmentation and vocabulary development).

## 2 Method

### 2.1 Participants

The data for the study were collected longitudinally from infants and their caregivers when the infants were 9, 15, and 21 months. Fifty English‐speaking families with infants in the target age range were originally recruited in a largely middle‐class urban community in Scotland through magazine advertisements, fliers, play groups, and word of mouth. One family withdrew from the study before the data collection began. Data from two other families were excluded from the study because the infants began to receive a substantial amount of non‐English input (Doric and Urdu, respectively) as the data collection progressed. The final pool of participants consisted of 47 infants (24 girls and 23 boys) and their families. All infants were born full‐term and had no known history of ear infections or hearing problems. Thirty‐three of the target infants were first‐born and 14 were later‐born. In all families, the mother was the primary caregiver and was a native speaker of English. The fathers of the infants were also native speakers of English with three exceptions (native speakers of Chichewa, German, and Russian, respectively), but English was the main language of communication in all families. Participating families received compensation in the form of gift vouchers.

### 2.2 Procedure

#### 2.2.1 Recording sessions

When the infant was 9 months of age, a researcher visited the family and provided them with a digital recorder that was set to record audio at 16‐bit/44.1 kHz in WAV format. Families were instructed to record verbal interactions with their infant that are representative of their daily routines. Most recordings contained interactions at meal‐time, free play, bath‐time, and bed‐time. The make‐up of interlocutors varied across sessions and families, ranging from interactions featuring only the infant and the mother, to those featuring siblings and grandparents. However, all families had samples of mother‐child interactions. Families were asked to make recordings that are at least 15 min long each, until they accumulated 90‐min worth of material. A recording log was provided on which participants recorded the date, length, context and interlocutors of each session. One of the authors transcribed 60 min of recording from each infant, using the CHAT format. These transcripts were then checked for accuracy and consistency by a research assistant, and finally by another author.

#### 2.2.2 Vocabulary development assessment

At each of the three data collection points (9, 15, and 21 months), parents of the infant were asked to complete a UK version of the MacArthur Communicative Development Inventory (CDI) (Alcock, Meints, & Rowland, 2017). Two modifications were made to the original UK CDI. First, we added several common words used in Scottish English (e.g., *aye, bairn*,* dinnae*, and *wee*). Second, as we were interested in the specific phonological shapes of the words learned by the infants, we also added alternative forms to the list. Thus, forms such as *doggy*,* piggy*, and *night‐night* were listed alongside *dog*,* pig*, and *goodnight* to be marked separately. If an alternative form used in the family was not listed, they were asked to write it down instead of ticking the equivalent form on the list (e.g., *mama* for *mummy*,* dindin* for *dinner*).

### 2.3 Measures

Measurement of input variables was carried out using the transcribed speech from the recordings. Since recent findings indicate that overheard linguistic input has limited impact on children's lexical development (Shneidman, Arroyo, Levine, & Goldin‐Meadow, 2013; Shneidman & Goldin‐Meadow, 2012; Weisleder & Fernald, 2013), the lexical input measures below did not include utterances that were clearly addressed to an interlocutor other than the infant (e.g., exchanges between the mother and father, or between the mother and a sibling of the target infant). Note that the lexical input measures could be overlapping, so that the same word could be counted as both onomatopoeic and reduplicated (e.g., *choo‐choo*) or both diminutive and reduplicated (e.g., *daddy*).

#### 2.3.1 Iconicity in lexical input

Two methods were used to measure iconicity in lexical input. First, we calculated the token‐based proportion of words addressed to the infant that were judged to be onomatopoeic in nature. These included items that describe sounds produced by animals (*woof*,* tweet‐tweet*,* meow*), objects (*tick‐tock*), and vehicles (*chugga‐chugga*,* vrrrm*), similar forms that are used referentially (e.g., *choo‐choo* as in “Do you want to play with your choo‐choo?”), and motion words used as related actions were performed (*bang*,* boing, splash*). Hereafter we call this measure onomatopoeia. The second measurement was based on the iconicity ratings of 3,001 English words reported in Winter, Perlman, Perry, and Lupyan (2017). The ratings were obtained by asking native speakers of English on Mechanical Turk to judge the iconicity of words on a scale ranging from −5 (‘words that sound like the opposite of what they mean’) to 5 (‘words that sound like what they mean’). In order to calculate the overall iconicity profile of the lexical input in our study, we first matched the words in Winter et al.'s database with those in the speech addressed to each of our infants. When iconicity ratings were available for specific morphological variants of the same lemma (e.g., *is, am, are* … for BE, *did, do, does* … for DO), they were matched with the same word forms in the infant‐addressed speech. Otherwise, we treated the entries in Winter et al. (2017) as lemmas, and applied the same ratings to various inflected forms in the input (e.g., the rating for DOG was used for *dog* and *dogs*). Overall, 80.6% of the word tokens in the 9‐month recordings could be matched this way. These were used to calculate the mean iconicity score for a given infant by multiplying each available rating by the token frequency of the corresponding word in the speech addressed to the infant, and then dividing the sum by the total token number of input words with iconicity ratings. Hereafter we refer to this measure as iconicity ratings.

#### 2.3.2 Diminutives in lexical input

As mentioned in the Introduction, diminutives are characterized by two structural properties: a fixed phonological edge (in English, a final /i/) and a regular prosodic form (in English, a disyllabic structure with initial stress). To capture both aspects in English IDS‐specific words, we operationalized diminutives as initially stressed disyllabic words ending in /i/. Because there is no evidence that infants at 9 months can distinguish ‘true’ diminutives (e.g., *doggy*,* footie, froggie*) from words that happen to have the same phonological structure (e.g., *soggy*,* baby, pretty*), our operationalization of diminutives included all words that met these phonological conditions regardless of their morphological structure or grammatical category. The input measure was the token‐based proportion of such words in the IDS at 9 months.

#### 2.3.3 Reduplication in lexical input

reduplication was operationalized as the token‐based proportion of words in the input at 9 months consisting only of (sets of) syllables that differ at most by the onset or rhyme. This definition applied to disyllabic words with two segmentally identical syllables (e.g., *night‐night*), disyllabic words with syllables differing only by the onset (e.g., *bow‐wow*) or rhyme (e.g., *tick‐tock, daddy*), and compounded disyllabic words with syllables differing only by the onset (e.g., *teeny‐weeny*,* easy‐peasy*).

#### 2.3.4 Vocabulary size estimates

Estimates of vocabulary size were calculated by summing up the number of words each child reportedly produced at 9, 15, and 21 months according to CDI responses. All the words in the CDI that were produced by at least one child in our sample population by 21 months are listed in the Supplementary Materials (S3).

#### 2.3.5 Control variables

Early vocabulary development is known to be influenced by the lexical diversity in the input, and relatedly, the socio‐economic status (SES) of the caregivers (Hart & Risley, 1995; Huttenlocher, Haight, Bryk, Seltzer, & Lyons, 1991; Rowe, 2008; 2012). To guard against the possibility that these factors confound effects from the characteristics of the individual words in IDS, we included measures of lexical diversity and caregiver educational level in our analysis. Lexical diversity was measured using moving average type‐token ratios (MATTR) of the IDS in the transcripts, following the method described in Covington and McFall (2010). We used a window size of 100 words, and calculated the ratio of the word types and word tokens for the first to 100th words, then for the second to 101st words, for words 3–102 and so on up to the end of each transcript. The mean of these ratios was recorded as the MATTR of that particular sample. We used the mother's highest educational degree as an index of the caregiver's SES, with a 4‐point scale ranging from 0 = no high school education, 1 = high school qualification, 2 = undergraduate degree, to 3 = postgraduate degree.

## 3 Results

### 3.1 General characteristics of infant‐directed speech

The infant‐directed speech in our 9‐month data showed characteristics that are typical of speech addressed to infants of this age. The mean length of utterance (MLU) was 4.73 words (*SD* = 3.47) for mothers and 4.03 words (*SD* = 3.10) for fathers, values similar to those reported in previous studies (Kavanaugh & Jirkovsky, 1982; Phillips, 1973; Soderstrom, Blossom, Foygel, & Morgan, 2008) and shorter than those in typical adult‐directed speech (for example, Phillips reports that the mean MLUs of parents of 8‐month‐olds are higher than eight words when they engage in adult‐to‐adult conversations). Another feature frequently identified in previous studies on IDS and also found in our corpus was a high incidence of yes‐no questions. Similar to the percentages reported in other studies (Kavanaugh & Jirkovsky, 1982; Newport, Gleitman, & Gleitman, 1977; Soderstrom et al., 2008), 22.7% of the utterances addressed to the infants were yes‐no questions.

As expected, there were many types of lexical items unique to the IDS register (i.e., baby‐talk words). Onomatopoeic items included sound‐symbolic words and expressions related to animals (e.g., *quack, moo, oink, ribbit, tweet, woof*), vehicles (e.g., *nee‐naw, chugga‐chugga, choo‐choo*), and objects (e.g., *ring‐ring, cha‐ching, tick‐tock*). As can be seen from these examples, many of these were reduplicated or could be in either nonreduplicated or reduplicated form (e.g., *quack* vs. *quack‐quack*). Others were only attested in nonreduplicated forms (e.g., *a‐tishoo, cock‐a‐doodle‐doo*). A number of morphologically transparent diminutives were present (e.g., *doggy, piggy, horsie, feetie, drinkie, sockie, truckie*), as well as less transparent ones (e.g., *bunny, tummy, teddy*). Reduplicated words included conventional forms (e.g., *wee‐wee, night‐night, din‐din, silly‐billy, teeny‐weeny, wibbly‐wobbly*), but also nonconventional productive forms (e.g., *soupie‐soupie, walk‐walk, food‐food, brush‐brush*). In addition, there were many verbal routines typical of IDS (e.g., *peekaboo, coochie‐coo, a‐boo, clippity‐clop*) and terms of endearment (e.g., *poppet, cheeky‐chops, sweetie‐pie*). While a range of such IDS‐unique items were identified, they did not constitute a large part of the lexical input in terms of token proportions (see the descriptive statistics in the next section). With the exception of the word *mummy* (*mommy*), the most frequent words in the input were predominantly function words and basic verbs.^2^

### 3.2 Descriptive data of predictors

Table 1 presents the mean and standard deviation of the predictor variables as well as the correlations between them. The overall token proportions of the words of interest in the input were fairly low, with onomatopoeic words in particular accounting for less than 1% of the lexical input on average. The means for diminutive and reduplicated lexical input were approximately 4% and 2%, respectively. Note that the proportions of diminutives and reduplicated words are based on the form‐based operationalizations given above and include not only nouns but also other forms such as adjectives (e.g., *soggy*,* easy*).

**Table 1:** Mean (*M*) and standard deviation (*SD*) of predictor variables and correlations between variables

	Mean (*SD*)	1	2	3	4	5
1. Onomatopoeia (%)	0.85 (0.62)					
2. Iconicity rating	0.68 (0.05)	.50***				
3. Diminutives (%)	4.09 (1.11)	.07	.24			
4. Reduplication (%)	1.89 (0.99)	.07	.32*	.61***		
5. MATTR	0.59 (0.03)	.05	−.04	−.02	−.22	
6. Mother's education	2.36 (0.74)	−.02	.02	.25	−.02	.14

*Note*. *p <* *.05, ***p <* *.001.

There was a significant correlation between the proportion of diminutives and the proportion of reduplicated words in the input (*r *=* *.61, *p *<* *.001). This is due to an overlap in these categories, with approximately 24% of word types with reduplication also matching our definition of diminutives (e.g., *baby, mummy, daddy, cookie, wee‐wee*). A significant positive correlation was also found between the proportion of onomatopoeic words and the iconicity rating of the input (*r *=* *.50, *p *<* *.001), indicating that, despite their very low token proportion in the input, onomatopoeic words affected the overall level of iconicity in the infant‐directed speech.^3^ Iconicity rating was also positively correlated with the proportion of reduplicated words in the input (*r *=* *0.32, *p *=* *.026), suggesting that reduplicated words tend to be perceived as more iconic. In contrast, there was no correlation between the proportions of onomatopoeic words and reduplicated words. This is likely due to the fact that even though many onomatopoeic words are reduplicated (29.4%), the vast majority of reduplicated words are not onomatopoeic (82.5%). No significant correlations were found between the lexical input measures and estimates of lexical diversity (MATTR) or mother's level of education.

### 3.3 Unconditional model of overall vocabulary growth

**Figure 1:**
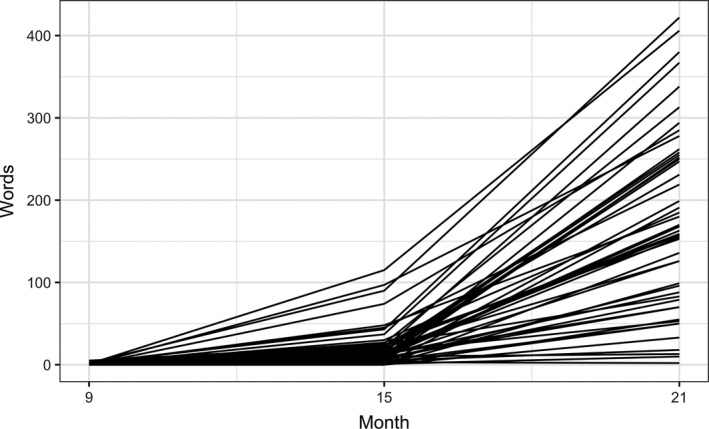
Change in overall productive vocabulary size. Each line represents an infant with its *y*‐axis value indicating the number of word types in his or her productive vocabulary. The type counts include onomatopoeic, diminutive, or reduplicated words.

In order to analyze how the overall growth of vocabulary size between 9 and 21 months is related to the lexical input characteristics at 9 months, we fitted two‐level growth models to our longitudinal data using the lmer function in R's lme4 package (Bates, Maechler, Bolker, & Walker, 2015). The first step was to select a baseline model with time terms only that best fits the trajectory of vocabulary size change in the individuals (see Fig. 1). The mean productive vocabulary size, based on the CDI responses, was 0.7 words (range: 0–5) at 9 months, 21.6 words (range: 0–115) at 15 months, and 176.5 words (range: 2–422) at 21 months.

The baseline model we selected was one with both linear and quadratic time terms as fixed effects, and it included random intercepts for both the linear and quadratic terms and random slopes for the linear term. The details of the model selection process can be found in the Supplementary Materials (Appendix S1). The parameter estimates of this model are summarized in Table 2 (Model 1). The estimated average size of productive vocabulary at 15 months, represented by the intercept, is 21.6 words. An accelerated increase in the vocabulary size between 9 and 21 months is indicated by significant linear and quadratic effects.

**Table 2:** Unconditional model (Model 1) and single‐factor conditional models of vocabulary size (all CDI words) with diminutives (Model 2), reduplication (Model 3), and maternal education (Model 4)

	Model 1	Model 2	Model 3	Model 4
Level 1				
Intercept	21.57** (7.04)	21.57** (6.63)	21.57** (6.73)	21.57** (6.75)
Age	14.65*** (1.36)	14.65*** (1.28)	14.65*** (1.30)	14.65*** (1.30)
Age^2^	1.86*** (0.13)	1.86*** (0.12)	1.86*** (0.12)	1.86*** (0.12)
Level 2				
Diminutives		5.94 (6.65)		
Diminutives × Age		3.17* (1.28)		
Diminutives × Age^2^		0.37** (0.12)		
Reduplication			5.76 (6.76)	
Reduplication × Age			2.72* (1.30)	
Reduplication × Age^2^			0.30* (0.12)	
Maternal education				5.85 (6.77)
Maternal education × Age				2.69* (1.30)
Maternal education × Age^2^				0.29* (0.12)
Deviance		1434.0**	1438.5*	1434.0**

*Notes*. *p <* *.05, **p <* *.01, ***p <* *.001. Deviance significance levels are based on comparison to the unconditional model (Model 1).

The next step in our analysis was to evaluate the individual contributions of the main variables of interest—onomatopoeia, iconicity rating, diminutives, and reduplication—by building a series of conditional growth models with each of these variables as an additional level‐2 predictor, and comparing them to the baseline model. We also used the same method to test the effects of lexical diversity and maternal education, which were extraneous to our focus. We then explored the combined effects of the significant predictors by examining models with two level‐2 predictors. All predictor variables were centered and scaled. Deviance significance levels of all conditional models were checked with a chi‐square test against the unconditional model. The *p*‐values for individual parameters were calculated from F statistics based on Satterthwaite's approximation of denominator degrees of freedom. For the sake of focus and readability, we only report the models with significant level‐2 predictors. The details of other models are reported in the Supplementary Materials (Appendix S2).

### 3.4 Conditional models of vocabulary growth with single level‐2 predictors

The growth models with one additional level‐2 predictor showed no significant effects of onomatopoeia or lexical diversity (MATTR). However, the proportion of diminutives in the lexical input was shown to have a positive effect on both the linear age parameter (*p *=* *.017) and quadratic age parameter (*p *=* *.003). Model 2 in Table 2 predicts that for every one standard deviation of increase in the proportion of diminutives (approximately 1% of the overall lexical input), the linear vocabulary growth increased by 3.17 words per month as well as being accelerated by 0.37 words per month squared. The proportion of reduplicated words in the lexical input also had a significant positive effect on both the linear age parameter (*p *=* *.042) and quadratic age parameter (*p *=* *.016). Model 3 in Table 2 predicts that for every one standard deviation of increase in the proportion of reduplication (i.e., approximately 1% of the overall lexical input) the linear vocabulary growth increased by 2.72 words per month as well as being accelerated by 0.30 words per month squared. Finally, maternal education had a positive effect on both the linear age parameter (*p *=* *.042) and quadratic age parameter (*p *=* *.016) (see Model 4 in Table 2). This shows that a higher level of maternal education was associated with a greater linear increase as well as a more accelerated growth of vocabulary.

### 3.5 Conditional models of vocabulary growth with two level‐2 predictors

Table 3 presents models examining the combined effects of the three predictors with significant effects in the single predictor models. In Model 5, the effects of maternal education on the linear growth parameter approached significance (*p *=* *.078), and diminutives had effects on the linear (*p *=* *.029) and quadratic (*p *=* *.013) growth parameters. In Model 6, education showed effects on the linear (*p *=* *.039) and quadratic (*p *=* *.025) growth parameters, and reduplication also had effects on the linear (*p *=* *.039) and quadratic (*p *=* *.019) growth parameters. Taken together, these results indicate that the proportions of diminutives and reduplication in the lexical input predict vocabulary growth even when the effects of maternal education are taken into account. In the final model (Model 7), we explored the independent effects of diminutives and reduplication on vocabulary size change. Because diminutives and reduplication were correlated (see Table 1), we residualized the measure of diminutives against the measure of reduplication to avoid collinearity. Residualized diminutives had effects on the quadratic parameter (*p *=* *.048) and reduplication had effects on both the linear (*p *=* *.029) and quadratic (*p *=* *.013) parameters. This model shows that diminutives and reduplication make independent contributions to the growth of vocabulary size.

**Table 3:** Two‐factor conditional models of vocabulary size (all CDI words) with maternal education and diminutives (Model 5), maternal education and reduplication (Model 6), and diminutives and reduplication (Model 7)

	Model 5	Model 6	Model 7
Level 1			
Intercept	19.89** (6.64)	21.60** (6.41)	21.57** (6.45)
Age	14.32*** (1.28)	14.66*** (1.23)	14.65*** (1.24)
Age^2^	1.86*** (0.12)	1.86*** (0.12)	1.86*** (0.12)
Level 2			
Maternal education	6.63 (6.96)	6.46 (7.16)	
Maternal education × Age	2.41† (1.34)	2.92* (1.37)	
Maternal education × Age^2^	0.21 (0.13)	0.31* (0.14)	
Diminutives	6.35 (6.84)		5.75 (8.32)
Diminutives × Age	2.97* (1.32)		2.82† (1.60)
Diminutives × Age^2^	0.32* (0.13)		0.31* (0.16)
Reduplication		6.41 (7.27)	6.23 (6.48)
Reduplication × Age		2.96* (1.40)	2.82* (1.25)
Reduplication × Age^2^		0.32* (0.14)	0.31* (0.12)
Deviance	1427.4*	1427.7*	1429.9*

*Notes*. ^†^ *p *<* *.1, *p <* *.05, **p <* *.01, ***p <* *.001. Proportion of diminutives is residualized against reduplication in Model 7. Deviance significance levels are based on comparison to the unconditional model (Model 1).

### 3.6 Growth models of vocabulary without baby‐talk features

The analysis presented above was based on estimates of the overall vocabulary size using all the words in the CDI reports. Among the words included in the vocabulary counts are items that carry the baby‐talk features used as predictors (i.e., onomatopoeia, diminutives, and reduplication). This means that the correlations we found between input features and vocabulary growth are partly due to lexical items included in both the predictors and the dependent variable in the models. For example, infants who hear more reduplicated words (e.g., *choo‐choo, daddy, teeny‐weeny*) may display faster increase in overall vocabulary size because these words themselves are more likely to be learned than nonreduplicated words. However, our hypothesis was that the effects of IDS‐specific lexical features in the input should extend *beyond* the acquisition of the very words that have those features and apply indirectly to other words, because learning baby‐talk (‐like) words should bootstrap vocabulary development in general. In order to test this particular aspect of our hypothesis, we reran the analysis using estimates of vocabulary without including words that have onomatopoeia, diminutives, or reduplication. Accordingly, our vocabulary size count in the analysis below excluded all words in the CDI category ‘Sounds’ (e.g., *choo‐choo, moo, quack*) except the words *uhoh* and *ouch*, and all words matching our definitions of diminutives and reduplication described above (e.g., *froggie, baby, night‐night, clap‐clap, tick‐tock*). The specific words excluded from this analysis are indicated in the word list in the Supplementary Materials (S3).

### 3.7 Unconditional model of vocabulary growth without words with baby‐talk features

**Figure 2:**
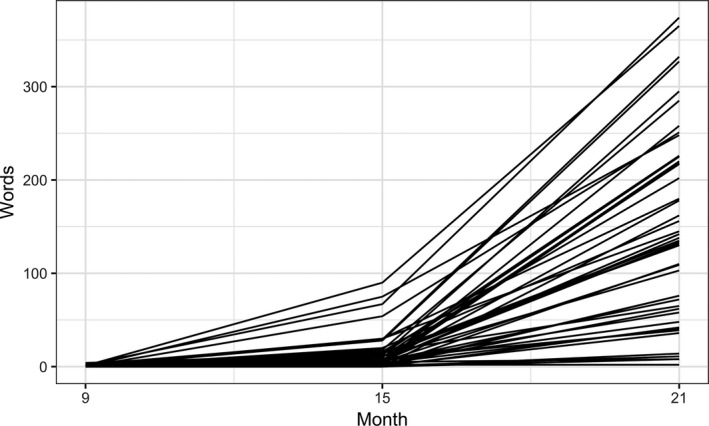
Change in productive vocabulary size. Each line represents an infant with its *y*‐axis value indicating the number of word types in his or her productive vocabulary. The type count does not include onomatopoeic, diminutive, or reduplicated words.

Fig. 2 shows the change in vocabulary size estimates excluding words that match definitions of onomatopoeia, diminutives or reduplication. The mean size of vocabulary was 0.4 words (range: 0–4) at 9 months, 14.5 words (range: 0–91) at 15 months, and 151.8 words (range: 2–374) at 21 months. An unconditional model using the same formula as that for Model 1 was applied to this dataset (Model 8 in Table 4). The adopted baseline model with linear and quadratic terms had a significantly better fit with the data than a linear‐only model (χ^2^(1) = 111.19, *p *<* *.001).

**Table 4:** Unconditional model (Model 8) and two‐factor conditional model of vocabulary size (excluding ‘baby‐talk words’) with diminutives and reduplication (Model 9)

	Model 8	Model 9
Level 1		
Intercept	14.38* (6.32)	14.38* (5.87)
Age	12.57*** (1.23)	12.57*** (1.14)
Age^2^	1.71*** (0.12)	1.71*** (0.11)
Level 2		
Diminutives		2.46 (5.92)
Diminutives × Age		2.19 (1.48)
Diminutives × Age^2^		0.29* (0.14)
Reduplication		4.67 (5.92)
Reduplication × Age		2.42* (1.15)
Reduplication × Age^2^		0.27* (0.11)
Deviance	1422.6	1405.4*

*Notes*. *p <* *.05, ***p <* *.001. Proportion of diminutives is residualized against reduplication. Deviance significance levels are based on comparison to the unconditional model (Model 8).

### 3.8 Conditional models of vocabulary growth without words with baby‐talk features

We followed the same model selection procedure as the analysis for the overall vocabulary growth. The details can be found in the Supplementary Materials (S2). The results confirmed that there were no significant contributions from onomatopoeia, iconicity rating, or MATTR as individual factors, while the proportion of diminutives and reduplicated words as well as maternal education all had a positive effect on both the linear age parameter in the single‐factor conditional models. In two‐factor conditional models, diminutives and reduplication had significant effects on the quadratic growth parameter and marginal effects on the linear growth parameter when combined with maternal education.

Most importantly, as shown in Table 4, when diminutives and reduplication were included in a two‐factor conditional model with diminutives residualized against reduplication, residualized diminutives had effects on the quadratic parameter (*p *=* *.043), and reduplication had effects on both the linear (*p *=* *.042) and quadratic (*p *=* *.015) parameters. Thus, even when we removed words carrying baby‐talk features from the vocabulary count, both diminutives and reduplication as lexical input factors predicted the trajectory of vocabulary size growth.

In general, these outcomes are fairly comparable to those obtained for the overall vocabulary growth. Vocabulary growth estimates without words matching definitions of onomatopoeia, diminutives, or reduplication were related to input measures of diminutives and reduplication, along with maternal education level. The results are therefore consistent with the hypothesis that certain ‘baby‐talk features’ of the lexical input to infants promote vocabulary growth beyond those words that contain the same lexical features.

## 4 Discussion

Unlike most documented differences between IDS and ADS, baby‐talk words or the register‐specific words in IDS present a unique set of linguistic items that are not part of the core repertoire of adult language use. Furthermore, they exhibit distinct referential and phonological characteristics such as iconicity, diminutives and reduplication, which are also untypical of lexical items in ADS. Rather than a peripheral peculiarity in the learner's linguistic environment, these register‐specific words may play an important role in infants’ early lexical development by providing referential insights, constraints on possible referents, and cues for word segmentation. Our specific prediction was that characteristics of baby‐talk words would accelerate word learning in the early stages of vocabulary development. We tested this hypothesis by examining whether the overall change of vocabulary size between 9 and 21 months is related to measures of iconicity, diminutives and reduplication in the lexical input that infants receive. The growth of productive vocabulary was related to measures of diminutive and reduplicated lexical input, although not to measures of iconicity. Overall, these findings can be interpreted as evidence that certain properties that are typically associated with register‐specific vocabulary used with young infants—at least diminutives and reduplication—do indeed facilitate general vocabulary development. The observed effects are particularly remarkable given that the proportions of words identified as having diminutive or reduplicated structures were not overwhelmingly large in the overall speech addressed to the infants (typically not more than 5%), and they highlight the potential impact a small section of the linguistic input can have on early language development.

The relationship between diminutive input and global lexical growth supports the claim made by Kempe et al. (2007a,b) that diminutives facilitate word segmentation by virtue of their regular prosodic structure and invariant endings. It is worth noting that our measure combined two characteristics of diminutive words: initially stressed disyllables and /i/ ending. We also performed additional exploratory analyses to tease apart these effects but found no independent effects of the prosodic structure and the word ending. The best interpretation of this finding therefore is that the observed effects come from the combination of prosodic cues and edge invariance. The association found between reduplication and lexical growth corroborates the experimental evidence that reduplicated words are more easily segmentable than nonreduplicated words by infants at 9 months (Ota & Skarabela, 2018). The current findings suggest that the presence of reduplicated words in the input can also facilitate general vocabulary development, presumably because once segmented and acquired, reduplicated words can be used to anchor further segmentation of fluent speech input.

One might wonder whether the associations found between diminutives, reduplication, and lexical size growth are largely due to appellations of parents (e.g., *mummy*,* daddy*), which meet the definitions of both diminutives and reduplication. Since such words are often acquired before 6 months, the correlations could be a by‐product of these individual words. Infants whose input contains frequent occurrences of *mummy* and *daddy* might be at advantage because they can use those words to segment incoming speech. However, this possibility is unlikely for two reasons. First, a follow‐up analysis using the proportion of parental appellations per se failed to replicate the results obtained with general operationalizations of diminutives and reduplication. Second, the analysis using both residualized diminutives and reduplication showed that the source of the effects could not be reduced to reduplicated diminutives only.

In contrast to the results for diminutives and reduplication, the study did not find a significant association between iconicity in the input and overall vocabulary growth. This outcome does not seem to be in accordance with the sizeable body of evidence that iconic form‐meaning mappings are more readily learned across the lifespan, and it could be due to several methodological factors. First, our measures of iconicity might not have fully captured the variance in the input. The token‐based proportion of onomatopoeic words in our samples was quite low, averaging below 1% of the input. With this level of frequency, a sample consisting of 60 min of recording may be too small to obtain an accurate estimate of the actual proportion of onomatopoeic input individual infants receive. Furthermore, the iconicity ratings adopted from Winter et al. (2017) may have influenced the outcomes at least for two reasons. One is that the ratings are based on adult intuitions, which are likely to be different from that of infants with limited prior lexical knowledge. Crucially, adult judges may not distinguish ‘absolute’ iconicity (where there is an inherent resemblance between the form and meaning, e.g., onomatopoeia) and ‘relative’ iconicity (where there is an analogical relationship between the form of a group of words and their meaning, e.g., /gl‐/ and light as in *glitter, glimmer,* and *glow*). Thus, adult raters may consider both of these to be cases of “words that sound like what they mean” and rate them highly on the iconicity scale, but young learners are unlikely to benefit from relative iconicity until they have acquired a sufficient number of relevant words that reveal the regularity in the sound‐meaning mappings. Another issue related to the use of this existing database of iconicity is that the ratings could not be matched with the entire input data, leaving 20% of the words in the input unrated for iconicity. It is possible that those IDS words not included in the list of iconicity‐rated words have different iconicity profiles from the words featured in the rating database. Further estimation errors of iconicity in the input were potentially introduced by our decision to lemmatize word forms when specific ratings for morphological variants were not available. This step might have underestimated the iconicity of diminutivized forms, for example, because forms such as *doggy* and *piggy* may be perceived as being more iconic due to the semantic connotation of the –*y* ending, but were given the same ratings as the base form (e.g., *dog* and *pig*) in our analysis. To allay this concern, we checked the iconicity ratings of all the words in Winter et al. (2017) that could be interpreted as diminutives (*belly, budgie, buggy, bunny, daddy, kitty, mommy, owie, potty,* and *tummy*). The mean rating of these words (0.904 on a scale from −5 to 5) did not differ from the mean rating of all the words in the database (0.915, one‐sample *t*(9) = 0.038, n.s.), providing no evidence that the iconicity of diminutives tends to be higher than other words. However, we note that most of the diminutivized forms included in this simple analysis (e.g., *kitty, tummy*) are lexically frozen, and it is possible that the iconicity of more morphologically transparent diminutives such as *doggy* and *piggy*, which are not included in Winter et al. (2017), might have been underestimated in our main analysis.

A related limitation of the current study is that the input measure was taken from only one temporal point in the development (9 months). This single‐point sampling may have missed the critical phase when iconic lexical input has detectable effects on vocabulary development. According to the sound‐symbolism bootstrapping hypothesis, one of the main benefits of sound symbolic words is that they reveal the referential nature of sound‐meaning mappings in words. Given evidence that 6‐month‐olds already have some knowledge of common words such as food items and body parts (Bergelson & Swingley, 2012), it could be that infants discover this fundamental nature of words prior to 9 months. On the other hand, it is also possible that the impact of iconic sound‐meaning becomes more apparent after 9 months as vocabulary acquisition picks up pace and learners face the challenge of referential indeterminacy more frequently. The available findings that are pertinent to this matter do not, however, present a clear picture. For example, Massaro and Perlman's (2017) corpus‐based investigation of lexical acquisition in English shows that the tendency for iconic words to be acquired preferentially decreases rapidly after 12 months. In contrast, a study of 8‐ to 30‐month‐old children learning British Sign Language found that the effect of iconicity became more prominent with age (Thompson et al., 2012). A similar study on American Sign Language, however, did not find any age‐related increase of iconicity effects (Caselli & Pyers, 2017).

With specific regards to onomatopoeic words, our inspection of the transcribed infant‐directed speech data has revealed an interesting, and potentially important, distinction about the linguistic contexts in which they are used. In some cases, onomatopoeia is used referentially as a noun (e.g., “That's where the choo‐choo goes”). But in the majority of cases, it is used as a sound effect (e.g., “Trains go ‘choo choo’!”). Although the latter type of use highlights the iconic nature of onomatopoeia, it does not convey the form‐meaning relationship that underlies labels. This indicates that the lexical status of onomatopoeic items in IDS is often different from that of most diminutives and (nononomatopoeic) reduplicated words, which are used just like any other canonical labels in this respect. It is not immediately clear whether the difference in the contexts of use makes onomatopoeic words more or less accessible for the infant, but it does raise the need to investigate this distinction and its impact on early word learning in future research.

The single‐point input measure and the naturalistic longitudinal data used in this study also mean that we need to exercise the usual caution about inferring causation from correlational statistics. In particular, our design does not allow explorations of potential bi‐directional or reciprocal effects between the caregiver input and vocabulary development. One can imagine various scenarios in which the linguistic input is adjusted in response to the perceived developmental level of the child. For example, parents may begin to use more diminutives when they detect that their child has reached a certain level of readiness for further lexical development. In order to fully differentiate this type of development‐to‐input effects from input‐to‐development effects, we need input measures from more than one temporal point (although the matter is far more complicated than simply comparing correlations between multiple input measures; see Richards, 1994 for a classic discussion of this problem). Some observations in our data, however, are more consistent with the input being the cause of the individual differences in the vocabulary size development than the other way around. For instance, if the 9‐month input is reflective of the developmental stage of the infants at 9 months, which, in turn, predicts the developmental trajectory toward 21 months, we expect there to be a relationship between the vocabulary sizes at 9 and 21 months. However, there is no correlation between the 9‐month and 21‐month CDI counts (*r* ^2^ = .002, *p *=* *.783). Furthermore, if the caregivers are making adjustments in anticipation on the projected lexical development, we expect infants with a larger vocabulary at 9 months to be receiving more diverse lexical input at the same point. Such a relationship is not found in the correlation between the moving‐average type‐token ratios of the input and vocabulary size estimates at 9 months (*r* ^2^ = .001, *p *=* *.821).

There are still many unanswered questions about the exact nature of the impact of lexical input on the overall growth of vocabulary in early language development. While some extraneous variables such as lexical diversity and maternal SES were included in the analysis and found not to be accountable for the effects of diminutive or reduplicative lexical input, there are other factors which might mediate these effects. For example, mothers who use more baby‐talk words, and hence more diminutives and reduplicated words, may also have a more engaging style of interaction with their infants, which in turn accelerates their lexical growth. Examining such links is beyond the scope of this paper, but can be pursued in future work by using some proxy of engagement level, such as number of conversational turns taken per time unit or number of interrogatives. Another possible mediating factor is the delivery of baby‐talk words. For instance, it has been reported that parents produce onomatopoeic words with heightened acoustic and prosodic salience (Laing, Vihman, & Keren‐Portnoy, 2017). It may be that baby‐talk words or words with related features have a larger impact on lexical development than other types of words partly because of the way they are produced in IDS rather than their inherent phonological characteristics.

Another interesting question to address in future research is whether the effects of diminutive or reduplicative lexical input continue beyond this age. There are reasons to believe that the effects do not last over many years. For one thing, parental use of certain baby‐talk words (such as diminutives) diminishes after the first year (Berko Gleason et al., 1994), and with that, their impact should decline too. More importantly, as children become more experienced in word learning, they will also gain a wider range of strategies to segment or learn sound‐meaning mappings of novel words. Even the same strategy such as the use of known words as lexical cues for further segmentation becomes less dependent on certain types of words such as baby‐talk words. As such, any advantages that baby‐talk words may have in early lexical development should fade out with age. In this sense, it is fitting to consider the role of baby‐talk words as a bootstrapping device, whose main contribution is to kick‐start the process of lexical learning.

In conclusion, the present study provides evidence from naturalistic longitudinal data that lexical input containing properties typically associated with IDS‐specific words – diminutives and reduplication in particular – is related to the overall rate of vocabulary size growth in infants between 9 and 21 months. The results are in line with those of experimental studies demonstrating the facilitative effects of diminutives and reduplication in laboratory‐based word segmentation and word learning tasks (Kempe et al., 2007a,b; Ota & Skarabela, 2016, 2018). Taken together, these findings lend further support to the general idea that there are developmental advantages associated with the characteristics frequently found in the unique vocabulary of IDS. Previous research on IDS has demonstrated that the linguistic environment surrounding the infant may be more conducive to language learning due to quantitative adjustments made to existing linguistic properties in the adult language (e.g., speech rate, pitch range, and sentence length). The current study suggests that language further accommodates the infant learner by introducing items that are not part and parcel of the adult system. Even though words such as *choo‐choo* and *bunny* appear superfluous, they may play an important role in bootstrapping the development of the lexicon as a whole.

## Supporting information


**Appendix S1.** Unconditional model of overall vocabulary growth.
**Appendix S2.** Conditional models of overall vocabulary growth.
**Appendix S3.** Words in the CDI questionnaire produced by at least one child.

## Notes

1 Given parallel findings from sign language, this statement can be extended to any type of referential insight in language—spoken or signed. That is, learners must discover that words in any modality map some signal (sounds/signs) with meaning.

2 The 50 most frequent words, in order of frequency, were as follows: *you, the, it, that, a, go, to, and, oh, we, do, what, your, get, are, is, I, there, on, one, have, can, in, good, want, this, yeah, up, mummy, look*,* right, for, where, come, no, like, here, of, see, OK, he, now, all, not, with, little, think, me, just, put, some*. These words made up 56.2% of the overall lexical input (by token count).

3 Onomatopoeia received a high rating in the iconicity database, with a mean of 3.28 (on a scale from −5 to +5) and range of 1.8 (*peekaboo*) to 4.08 (*quack*).
