# Supplementary material for: Why Choo‐Choo Is Better Than Train: The Role of Register‐Specific Words in Early Vocabulary Growth
Source: Cogn Sci. 2018 Jul 11;42(6):1974–99. doi: 10.1111/cogs.12628 (PMC6120503; doi:10.1111/cogs.12628)
Supplement: Supplementary file 1 — Appendix S1. Unconditional model of overall vocabulary growth. Appendix S2. Conditional models of overall vocabulary growth. Appendix S3. Words in the CDI questionnaire produced by at least one child. [file COGS-42-1974-s001.docx]

**Why *choochoo* is better than *train*:**

**The role of register-specific words in early vocabulary growth**

**Supplementary materials**

Mitsuhiko Ota, Nicola Davies-Jenkins, & Barbora Skarabela

University of Edinburgh

**Appendix S1. Unconditional model of overall vocabulary growth**

This section outlines the procedure we followed in order to select the unconditional model for overall vocabulary growth. As the observed pattern of vocabulary growth in Figure 1 suggested a nonlinear trend, we first examined the fit of models with both linear and quadratic terms. Because a full quadratic model that includes random intercepts and random individual slopes for both the linear and quadratic terms failed to identify the random effect parameters, we compared two one-drop quadratic models, one without linear random slopes and one without quadratic random slopes, and found that the variance was much smaller for the quadratic random slopes (0.54) than for the linear random slopes (77.58). The quadratic model without the quadratic random slopes also had a better fit than a model with a linear time term only (χ^2^(1) = 111.19, *p* < .001), and it was adopted as our baseline model of unconditional growth. The formulation of the baseline model is given below, where *Y_ij_* is the number of words in the productive vocabulary at month *i* for infant *j*, the intercept *β*_0_*_j_* represents the estimated number of words for *j* at the centered age (15 months), the linear parameter *β*_1_*_j_* represents rate of change in vocabulary size for *j*, and the quadratic parameter *β*_2_*_j_* represents acceleration/deceleration of vocabulary growth for *j*. The intercept and linear parameter included error terms (*U*_0_*_j_* and *U*_1_*_j_*) but the quadratic parameter was modelled on the average estimate only.

Level 1

*Y_ij_* = *β*_0_*_j_* + *β*_1_*_j_*age*_ij_* + *β*_2_*_j_*age^2^*_ij_* + *R_ij_* (1)

Level 2

*β*_0_*_j_* = γ_00_ + *U*_0_*_j_* (2)

*β*_1_*_j_* = γ_10_ + *U*_1_*_j_* (3)

*β*_2_*_j_* = γ_20_ (4)

**Appendix S2. Conditional models of overall vocabulary growth**

The tables below report the models of vocabulary growth that were not included in the main text.

Table S2.1

*Single-factor conditional models of vocabulary size (all CDI words) with onomatopoeia (Model A), iconicity ratings (Model B), and MATTR (Model C).*

|  | Model A | Model B | Model C |
| --- | --- | --- | --- |
| Level 1 |  |  |  |
| Intercept | 21.57**  (7.03) | 21.57**  (7.03) | 21.57**  (6.84) |
| Age | 14.65***  (1.36) | 14.65***  (1.36) | 14.65***  (1.32) |
| Age^2^ | 1.86***  (0.13) | 1.86***  (0.13) | 1.86***  (0.13) |
| Level 2 |  |  |  |
| Onomatopoeia | 3.00  (7.06) |  |  |
| Onomatopoeia × Age | -0.46  (1.36) |  |  |
| Onomatopoeia × Age^2^ | -0.15  (0.13) |  |  |
| Iconicity rating |  | 4.42  (7.06) |  |
| Iconicity rating × Age |  | 0.11  (1.36) |  |
| Iconicity rating × Age^2^ |  | -0.09  (0.13) |  |
| MATTR |  |  | -7.99  (6.87) |
| MATTR × Age |  |  | -2.12  (1.32) |
| MATTR × Age^2^ |  |  | -0.14  (0.13) |
| Deviance | 1447.2, n.s. | 1448.1, n.s. | 1444.6, n.s. |

Notes: † *p* < .1, * *p* < .05, ** *p* < .01, *** *p* < .001. Deviance significance levels are based on comparison to the unconditional model (Model 1 reported in the main text).

Table S2.2

*Unconditional model (Model D) and single-factor conditional models of vocabulary size (excluding ‘baby-talk words’) with onomatopoeia (Model E), iconicity ratings (Model F), diminutives (Model F) and reduplication (Model H).*

|  | Model D | Model E | Model F | Model G | Model H |
| --- | --- | --- | --- | --- | --- |
| Level 1 |  |  |  |  |  |
| Intercept | 14.38*  (6.32) | 14.38*  (6.30) | 14.38*  (6.30) | 14.38*  (6.03) | 14.38*  (6.08) |
| Age | 12.57***  (1.23) | 12.57***  (1.23) | 12.57***  (1.23) | 12.57***  (1.17) | 12.57***  (1.19) |
| Age^2^ | 1.71***  (0.12) | 1.71***  (0.12) | 1.71***  (0.12) | 1.71***  (0.11) | 1.71***  (0.11) |
| Level 2 |  |  |  |  |  |
| Onomatopoeia |  | 1.93  (6.32) |  |  |  |
| Onomatopoeia × Age |  | -0.59  (1.23) |  |  |  |
| Onomatopoeia × Age^2^ |  | -0.14  (0.12) |  |  |  |
| Iconicity rating |  |  | 2.59  (6.33) |  |  |
| Iconicity rating × Age |  |  | 0.07  (1.24) |  |  |
| Iconicity rating × Age^2^ |  |  | -0.05  (0.12) |  |  |
| Diminutives |  |  |  | 3.28  (6.05) |  |
| Diminutives × Age |  |  |  | 2.55*  (1.18) |  |
| Diminutives × Age^2^ |  |  |  | 0.33**  (0.11) |  |
| Reduplication |  |  |  |  | 4.07  (6.11) |
| Reduplication × Age |  |  |  |  | 2.26†  (1.19) |
| Reduplication × Age^2^ |  |  |  |  | 0.27*  (0.11) |
| Deviance | 1422.6 | 1420.7, n.s. | 1422.2, n.s. | 1409.5** | 1413.5* |

Notes: † *p* < .1, * *p* < .05, ** *p* < .01, *** *p* < .001. Deviance significance levels are based on comparison to the unconditional model (Model D).

Table S2.3

*Single-factor conditional models of vocabulary size (excluding ‘baby-talk words’) with MATTR (Model I) and maternal education (Model J)*

|  | Model I | Model J |
| --- | --- | --- |
| Level 1 |  |  |
| Intercept | 14.38*  (6.15) | 14.38*  (6.05) |
| Age | 12.57***  (1.20) | 12.57***  (1.18) |
| Age^2^ | 1.71***  (0.12) | 1.71***  (0.11) |
| Level 2 |  |  |
| MATTR | -5.91  (6.17) |  |
| MATTR × Age | -1.85  (1.21) |  |
| MATTR × Age^2^ | -0.15  (0.12) |  |
| Maternal education |  | 4.39  (6.07) |
| Maternal education × Age |  | 2.45*  (1.18) |
| Maternal education × Age^2^ |  | 0.29*  (0.11) |
| Deviance | 1418.1, n.s. | 1412.0* |

Notes: † *p* < .1, * *p* < .05, ** *p* < .01, *** *p* < .001. Deviance significance levels are based on comparison to the unconditional model (Model D).

Table S2.4

*Multiple-factor conditional models of vocabulary size (excluding ‘baby-talk words’) with maternal education and diminutives (Model K), and maternal education and reduplication (Model L).*

|  | Model K | Model L |
| --- | --- | --- |
| Level 1 |  |  |
| Intercept | 13.19*  (6.02) | 14.47*  (5.79) |
| Age | 12.30***  (1.17) | 12.56***  (1.13) |
| Age^2^ | 1.70***  (0.11) | 1.70***  (0.11) |
| Level 2 |  |  |
| Maternal education | 5.23  (6.30) | 5.36  (6.46) |
| Maternal education × Age | 2.25†  (1.22) | 2.50†  (1.26) |
| Maternal education × Age^2^ | 0.23†  (0.12) | 0.27*  (0.12) |
| Diminutives | 3.93  (6.36) |  |
| Diminutives × Age | 2.42†  (1.24) |  |
| Diminutives × Age^2^ | 0.29*  (0.12) |  |
| Reduplication |  | 5.09  (6.53) |
| Reduplication × Age |  | 2.31†  (1.27) |
| Reduplication × Age^2^ |  | 0.25*  (0.12) |
| Deviance | 1401.9* | 1401.2* |

Notes: † *p* < .1, * *p* < .05, ** *p* < .01, *** *p* < .001. Deviance significance levels are based on comparison to the unconditional model (Model D).

S3. Words in the CDI questionnaire produced by at least one child. Items matching our operationalizations of onomatopoeia (Onom.), diminutives (Dim.) and/or reduplication (Redup.) are indicated by ‘x’ in the relevant column.

|  |  |  |  |  | Number of infants acquired | | |
| --- | --- | --- | --- | --- | --- | --- | --- |
| Category | Word | Onom. | Dim. | Redup. | 9 mo. | 15 mo. | 21 mo. |
| routines | *aye* |  |  |  | 0 | 0 | 1 |
| routines | *bath* |  |  |  | 0 | 10 | 37 |
| routines | *breakfast* |  |  |  | 0 | 0 | 22 |
| routines | *brush teeth* |  |  |  | 0 | 4 | 22 |
| routines | *bye* |  |  |  | 0 | 17 | 33 |
| routines | *bye bye* |  |  | x | 0 | 18 | 40 |
| routines | *call on phone* | |  |  | 0 | 1 | 21 |
| routines | *cheerio* |  |  |  | 0 | 0 | 1 |
| routines | *clap hands* |  |  |  | 0 | 1 | 25 |
| routines | *dinner* |  |  |  | 0 | 1 | 17 |
| routines | *don't* |  |  |  | 0 | 0 | 14 |
| routines | *goodbye* |  |  |  | 0 | 0 | 8 |
| routines | *goodnight* |  |  |  | 0 | 0 | 5 |
| routines | *hello* |  |  |  | 0 | 5 | 31 |
| routines | *hi* |  |  |  | 1 | 11 | 25 |
| routines | *hiya* |  |  |  | 4 | 22 | 32 |
| routines | *hush* |  |  |  | 0 | 0 | 2 |
| routines | *lunch* |  |  |  | 0 | 1 | 13 |
| routines | *music* |  |  |  | 0 | 0 | 12 |
| routines | *nap* |  |  |  | 0 | 1 | 15 |
| routines | *night-night* |  |  | x | 0 | 4 | 33 |
| routines | *no* |  |  |  | 1 | 13 | 42 |
| routines | *oops* |  |  |  | 0 | 6 | 27 |
| routines | *pee* |  |  |  | 0 | 1 | 10 |
| routines | *pee-pee* |  |  | x | 0 | 2 | 17 |
| routines | *peekaboo* |  |  |  | 0 | 9 | 27 |
| routines | *peepo* |  |  | x | 0 | 3 | 13 |
| routines | *please* |  |  |  | 0 | 3 | 30 |
| routines | *poo* |  |  |  | 0 | 5 | 25 |
| routines | *poo-poo* |  |  | x | 0 | 2 | 21 |
| routines | *sorry* |  |  |  | 0 | 1 | 22 |
| routines | *ssh* |  |  |  | 0 | 9 | 27 |
| routines | *supper* |  |  |  | 0 | 0 | 2 |
| routines | *ta* |  |  |  | 0 | 17 | 24 |
| routines | *tea* |  |  |  | 0 | 0 | 11 |
| routines | *thank you* |  |  |  | 0 | 6 | 27 |
| routines | *this little piggy* | |  |  | 0 | 0 | 4 |
| routines | *wait* |  |  |  | 0 | 0 | 10 |

S3. CDI words (continued)

|  |  |  |  |  | Number of infants acquired | | |
| --- | --- | --- | --- | --- | --- | --- | --- |
| Category | Word | Onom. | Dim. | Redup. | 9 mo. | 15 mo. | 21 mo. |
| routines | *want to* |  |  |  | 0 | 0 | 7 |
| routines | *wee* |  |  |  | 0 | 0 | 9 |
| routines | *wee wee* |  |  | x | 0 | 1 | 14 |
| routines | *yeah* |  |  |  | 0 | 5 | 19 |
| routines | *yes* |  |  |  | 0 | 5 | 39 |
| sounds | *baa baa* | x |  | x | 2 | 24 | 41 |
| sounds | *choo-choo* | |  | x | x | 0 | 6 |
| sounds | *cockadoodledoo* | x |  |  | 0 | 4 | 14 |
| sounds | *grr* | x |  |  | 1 | 18 | 35 |
| sounds | *meow* | x |  |  | 0 | 15 | 36 |
| sounds | *moo* | x |  |  | 1 | 21 | 43 |
| sounds | *ouch* |  |  |  | 0 | 1 | 30 |
| sounds | *quack* | x |  |  | 0 | 19 | 36 |
| sounds | *twee-tweet* | x |  | x | 0 | 7 | 26 |
| sounds | *uhoh* |  |  | x | 0 | 23 | 40 |
| sounds | *vroom* | x |  |  | 1 | 9 | 31 |
| sounds | *woof* | x |  |  | 0 | 23 | 43 |
| sounds | *yum* | x |  |  | 2 | 13 | 34 |
| animals | *animal* |  |  |  | 0 | 1 | 18 |
| animals | *bear* |  |  |  | 0 | 3 | 31 |
| animals | *beastie* |  | x |  | 0 | 0 | 4 |
| animals | *bee* |  |  |  | 0 | 2 | 32 |
| animals | *bird* |  |  |  | 0 | 5 | 22 |
| animals | *birdie* |  | x |  | 0 | 6 | 25 |
| animals | *bug* |  |  |  | 0 | 0 | 9 |
| animals | *bunny* |  | x |  | 0 | 3 | 21 |
| animals | *butterfly* |  |  |  | 0 | 1 | 22 |
| animals | *cat* |  |  |  | 0 | 10 | 37 |
| animals | *chicken* |  |  |  | 0 | 2 | 23 |
| animals | *cow* |  |  |  | 0 | 2 | 29 |
| animals | *dog* |  |  |  | 2 | 8 | 27 |
| animals | *doggy* |  | x |  | 0 | 9 | 30 |
| animals | *donkey* |  | x |  | 0 | 0 | 11 |
| animals | *duck* |  |  |  | 0 | 16 | 36 |
| animals | *duckie* |  | x |  | 0 | 3 | 9 |
| animals | *elephant* |  |  |  | 0 | 1 | 26 |
| animals | *fish* | |  |  | 0 | 7 | 29 |
| animals | *fishie* |  | x |  | 0 | 3 | 13 |
| animals | *fly* |  |  |  | 0 | 0 | 8 |
| animals | *frog* |  |  |  | 0 | 1 | 22 |
| animals | *froggie* |  | x |  | 0 | 1 | 15 |
| animals | *giraffe* |  |  |  | 0 | 3 | 22 |

S3. CDI words (continued)

|  |  |  |  |  | Number of infants acquired | | |
| --- | --- | --- | --- | --- | --- | --- | --- |
| Category | Word | Onom. | Dim. | Redup. | 9 mo. | 15 mo. | 21 mo. |
| animals | *horse* |  |  |  | 0 | 1 | 18 |
| animals | *horsie* |  | x |  | 0 | 0 | 24 |
| animals | *kitten* |  |  |  | 0 | 1 | 6 |
| animals | *kitty* |  | x |  | 0 | 0 | 6 |
| animals | *lamb* |  |  |  | 0 | 0 | 7 |
| animals | *lion* |  |  |  | 0 | 2 | 22 |
| animals | *monkey* | | x |  |  | 0 | 2 |
| animals | *mouse* |  |  |  | 0 | 1 | 24 |
| animals | *owl* |  |  |  | 0 | 2 | 30 |
| animals | *penguin* |  |  |  | 0 | 1 | 21 |
| animals | *pig* |  |  |  | 0 | 1 | 25 |
| animals | *piggie* |  | x |  | 0 | 1 | 14 |
| animals | *puppy* |  | x | x | 0 | 2 | 12 |
| animals | *pussy* |  | x |  | 0 | 0 | 5 |
| animals | *rabbit* |  |  |  | 0 | 2 | 22 |
| animals | *sheep* |  |  |  | 0 | 2 | 24 |
| animals | *snake* |  |  |  | 0 | 3 | 22 |
| animals | *spider* |  |  |  | 0 | 1 | 21 |
| animals | *squirrel* |  |  |  | 0 | 2 | 12 |
| animals | *tiger* |  |  |  | 0 | 2 | 18 |
| body | *arm* |  |  |  | 0 | 0 | 21 |
| body | *back* |  |  |  | 0 | 0 | 18 |
| body | *belly* |  | x |  | 0 | 1 | 13 |
| body | *belly button* |  |  |  | 0 | 0 | 16 |
| body | *bottom* |  |  |  | 0 | 1 | 15 |
| body | *bum* |  |  |  | 0 | 1 | 23 |
| body | *buttocks* |  |  |  | 0 | 0 | 2 |
| body | *cheek* |  |  |  | 0 | 1 | 20 |
| body | *chin* |  |  |  | 0 | 0 | 23 |
| body | *ear* |  |  |  | 0 | 2 | 37 |
| body | *eye* |  |  |  | 0 | 4 | 40 |
| body | *face* |  |  |  | 0 | 0 | 15 |
| body | *feet* |  |  |  | 0 | 0 | 28 |
| body | *finger* |  |  |  | 0 | 1 | 23 |
| body | *footsie* |  | x |  | 0 | 1 | 2 |
| body | *hair* |  |  |  | 0 | 2 | 33 |
| body | *hand* | |  |  | 0 | 1 | 35 |
| body | *head* |  |  |  | 0 | 2 | 27 |
| body | *knee* |  |  |  | 0 | 1 | 26 |
| body | *leg* |  |  |  | 0 | 0 | 20 |
| body | *lips* |  |  |  | 0 | 0 | 12 |
| body | *mouth* |  |  |  | 0 | 0 | 26 |

S3. CDI words (continued)

|  |  |  |  |  | Number of infants acquired | | |
| --- | --- | --- | --- | --- | --- | --- | --- |
| Category | Word | Onom. | Dim. | Redup. | 9 mo. | 15 mo. | 21 mo. |
| body | *nose* |  |  |  | 0 | 6 | 39 |
| body | *stomach* |  |  |  | 0 | 0 | 6 |
| body | *teeth* |  |  |  | 0 | 3 | 33 |
| body | *toe* |  |  |  | 0 | 2 | 28 |
| body | *tongue* |  |  |  | 0 | 0 | 15 |
| body | *tummy* |  | x |  | 0 | 1 | 26 |
| body | *tummy button* | |  |  | 0 | 0 | 7 |
| clothes | *bib* |  |  |  | 0 | 1 | 23 |
| clothes | *boots* |  |  |  | 0 | 1 | 26 |
| clothes | *button* |  |  |  | 0 | 4 | 23 |
| clothes | *cardigan* |  |  |  | 0 | 0 | 10 |
| clothes | *coat* |  |  |  | 0 | 1 | 25 |
| clothes | *dress* |  |  |  | 0 | 0 | 13 |
| clothes | *glasses* |  |  |  | 0 | 1 | 20 |
| clothes | *hat* |  |  |  | 0 | 5 | 40 |
| clothes | *jacket* |  |  |  | 0 | 1 | 21 |
| clothes | *jimjams* |  |  | x | 0 | 0 | 6 |
| clothes | *jumper* |  |  |  | 0 | 0 | 15 |
| clothes | *nappy* |  | x |  | 0 | 4 | 32 |
| clothes | *pants* |  |  |  | 0 | 0 | 13 |
| clothes | *PJs* |  |  |  | 0 | 0 | 3 |
| clothes | *pyjamas* |  |  |  | 0 | 0 | 14 |
| clothes | *scarf* |  |  |  | 0 | 0 | 13 |
| clothes | *shoes* |  |  |  | 0 | 17 | 42 |
| clothes | *sock* |  |  |  | 0 | 5 | 37 |
| clothes | *sockie* |  | x |  | 0 | 1 | 4 |
| clothes | *specs* |  |  |  | 0 | 0 | 2 |
| clothes | *trousers* |  |  |  | 0 | 0 | 20 |
| clothes | *vest* |  |  |  | 0 | 0 | 13 |
| clothes | *zip* |  |  |  | 0 | 1 | 14 |
| food | *apple* |  |  |  | 0 | 4 | 37 |
| food | *banana* |  |  |  | 1 | 16 | 42 |
| food | *biscuit* |  |  |  | 0 | 3 | 30 |
| food | *bread* |  |  |  | 0 | 1 | 27 |
| food | *butter* |  |  |  | 0 | 0 | 17 |
| food | *cake* |  |  |  | 0 | 2 | 32 |
| food | *carrots* | |  |  | 0 | 0 | 28 |
| food | *cereal* |  |  |  | 0 | 0 | 20 |
| food | *cheese* |  |  |  | 0 | 8 | 35 |
| food | *chicken* |  |  |  | 0 | 2 | 19 |
| food | *chips* |  |  |  | 0 | 1 | 16 |
| food | *chocolate* |  |  |  | 0 | 1 | 18 |

S3. CDI words (continued)

|  |  |  |  |  | Number of infants acquired | | |
| --- | --- | --- | --- | --- | --- | --- | --- |
| Category | Word | Onom. | Dim. | Redup. | 9 mo. | 15 mo. | 21 mo. |
| food | *coffee* |  | x |  | 0 | 0 | 10 |
| food | *cuppa* |  |  |  | 0 | 0 | 4 |
| food | *drink* |  |  |  | 0 | 2 | 20 |
| food | *egg* |  |  |  | 0 | 0 | 23 |
| food | *fish* |  |  |  | 0 | 1 | 26 |
| food | *food* |  |  |  | 0 | 1 | 15 |
| food | *ice-cream* | |  |  | 0 | 1 | 14 |
| food | *jam* |  |  |  | 0 | 1 | 15 |
| food | *juice* |  |  |  | 0 | 4 | 28 |
| food | *meat* |  |  |  | 0 | 0 | 3 |
| food | *milk* |  |  |  | 0 | 8 | 37 |
| food | *orange* |  |  |  | 0 | 2 | 27 |
| food | *pasta* |  |  |  | 0 | 3 | 30 |
| food | *peas* |  |  |  | 0 | 3 | 30 |
| food | *pizza* |  |  |  | 0 | 3 | 19 |
| food | *potato* |  |  |  | 0 | 2 | 21 |
| food | *sandwich* |  |  |  | 0 | 0 | 20 |
| food | *sausages* |  |  |  | 0 | 1 | 20 |
| food | *spread* |  |  |  | 0 | 0 | 1 |
| food | *tattie* |  | x | x | 0 | 0 | 2 |
| food | *tea* |  |  |  | 0 | 2 | 34 |
| food | *toast* |  |  |  | 0 | 5 | 32 |
| food | *water* |  |  |  | 0 | 3 | 33 |
| food | *yoghurt* |  |  |  | 0 | 2 | 32 |
| inside | *bath* |  |  |  | 0 | 13 | 38 |
| inside | *bathroom* |  |  |  | 0 | 1 | 12 |
| inside | *bathtub* |  |  |  | 0 | 0 | 2 |
| inside | *bed* |  |  |  | 0 | 1 | 32 |
| inside | *bedroom* |  |  |  | 0 | 0 | 16 |
| inside | *chair* |  |  |  | 0 | 1 | 28 |
| inside | *cooker* |  |  | x | 0 | 0 | 6 |
| inside | *cot* |  |  |  | 0 | 0 | 17 |
| inside | *couch* |  |  |  | 0 | 0 | 5 |
| inside | *door* |  |  |  | 0 | 4 | 36 |
| inside | *downstairs* |  |  |  | 0 | 0 | 12 |
| inside | *drawer* |  |  |  | 0 | 0 | 7 |
| inside | *fridge* | |  |  | 0 | 0 | 10 |
| inside | *highchair* |  |  |  | 0 | 0 | 11 |
| inside | *kitchen* |  |  |  | 0 | 0 | 19 |
| inside | *living room* |  |  |  | 0 | 0 | 6 |
| inside | *loo* |  |  |  | 0 | 0 | 4 |
| inside | *oven* |  |  |  | 0 | 0 | 10 |

S3. CDI words (continued)

|  |  |  |  |  | Number of infants acquired | | |
| --- | --- | --- | --- | --- | --- | --- | --- |
| Category | Word | Onom. | Dim. | Redup. | 9 mo. | 15 mo. | 21 mo. |
| inside | *settee* |  | x |  | 0 | 0 | 1 |
| inside | *sink* |  |  |  | 0 | 0 | 8 |
| inside | *sofa* |  |  |  | 0 | 0 | 9 |
| inside | *stairs* |  |  |  | 0 | 0 | 27 |
| inside | *table* |  |  |  | 0 | 1 | 20 |
| inside | *television* |  |  |  | 0 | 0 | 6 |
| inside | *telly* | | x |  | 0 | 1 | 13 |
| inside | *toilet* |  |  |  | 0 | 0 | 18 |
| inside | *TV* |  | x | x | 0 | 0 | 11 |
| inside | *upstairs* |  |  |  | 0 | 0 | 15 |
| inside | *window* |  |  |  | 0 | 1 | 19 |
| kinship | *brother* |  |  |  | 0 | 2 | 10 |
| kinship | *da* |  |  |  | 2 | 8 | 4 |
| kinship | *dad* |  |  |  | 1 | 7 | 17 |
| kinship | *daddy* |  | x | x | 0 | 21 | 43 |
| kinship | *grandma* |  |  |  | 0 | 2 | 14 |
| kinship | *granny* |  | x |  | 0 | 7 | 23 |
| kinship | *lassie* |  | x |  | 0 | 0 | 1 |
| kinship | *ma* |  |  |  | 2 | 5 | 6 |
| kinship | *mam* |  |  |  | 1 | 2 | 3 |
| kinship | *mother* |  |  |  | 0 | 0 | 1 |
| kinship | *mum* |  |  |  | 2 | 8 | 20 |
| kinship | *mummy* |  | x | x | 0 | 15 | 40 |
| kinship | *nan* |  |  |  | 0 | 1 | 7 |
| kinship | *own name* |  |  |  | 0 | 2 | 29 |
| kinship | *sister* |  |  |  | 0 | 0 | 8 |
| kinship | *uncle* |  |  |  | 0 | 0 | 10 |
| objects | *bag* |  |  |  | 0 | 5 | 32 |
| objects | *bin* |  |  |  | 0 | 1 | 23 |
| objects | *bottle* |  |  |  | 0 | 2 | 20 |
| objects | *bowl* |  |  |  | 0 | 2 | 25 |
| objects | *box* |  |  |  | 0 | 1 | 17 |
| objects | *broom* |  |  |  | 0 | 0 | 4 |
| objects | *brush* |  |  |  | 0 | 0 | 20 |
| objects | *bucket* |  |  |  | 0 | 0 | 13 |
| objects | *clock* |  |  |  | 0 | 1 | 14 |
| objects | *comb* | |  |  | 0 | 0 | 1 |
| objects | *computer* |  |  |  | 0 | 0 | 7 |
| objects | *cup* |  |  |  | 0 | 4 | 27 |
| objects | *dummy* |  | x |  | 0 | 2 | 11 |
| objects | *fork* |  |  |  | 0 | 0 | 24 |
| objects | *glass* |  |  |  | 0 | 0 | 12 |

S3. CDI words (continued)

|  |  |  |  |  | Number of infants acquired | | |
| --- | --- | --- | --- | --- | --- | --- | --- |
| Category | Word | Onom. | Dim. | Redup. | 9 mo. | 15 mo. | 21 mo. |
| objects | *hoover* |  |  |  | 0 | 2 | 11 |
| objects | *key* |  |  |  | 0 | 5 | 30 |
| objects | *lamp* |  |  |  | 0 | 0 | 7 |
| objects | *light* |  |  |  | 0 | 2 | 25 |
| objects | *medicine* |  |  |  | 0 | 0 | 10 |
| objects | *mobile* |  |  |  | 0 | 0 | 2 |
| objects | *money* | | x |  | 0 | 1 | 12 |
| objects | *paper* |  |  | x | 0 | 0 | 17 |
| objects | *phone* |  |  |  | 0 | 0 | 29 |
| objects | *picture* |  |  |  | 0 | 1 | 20 |
| objects | *pillow* |  |  |  | 0 | 0 | 13 |
| objects | *plant* |  |  |  | 0 | 0 | 8 |
| objects | *plate* |  |  |  | 0 | 0 | 15 |
| objects | *purse* |  |  |  | 0 | 0 | 8 |
| objects | *radio* |  |  |  | 0 | 0 | 6 |
| objects | *rubbish* |  |  |  | 0 | 0 | 8 |
| objects | *scissors* |  |  |  | 0 | 0 | 5 |
| objects | *soap* |  |  |  | 0 | 0 | 15 |
| objects | *spoon* |  |  |  | 0 | 2 | 31 |
| objects | *telephone* |  |  |  | 0 | 0 | 4 |
| objects | *toothbrush* |  |  |  | 0 | 3 | 21 |
| objects | *towel* |  |  |  | 0 | 0 | 21 |
| objects | *watch* |  |  |  | 0 | 0 | 12 |
| outside | *flower* |  |  |  | 0 | 4 | 24 |
| outside | *garden* |  |  |  | 0 | 1 | 19 |
| outside | *grass* |  |  |  | 0 | 0 | 13 |
| outside | *house* |  |  |  | 0 | 0 | 24 |
| outside | *moon* |  |  |  | 0 | 0 | 32 |
| outside | *outside* |  |  |  | 0 | 0 | 14 |
| outside | *park* |  |  |  | 0 | 1 | 26 |
| outside | *party* |  | x |  | 0 | 0 | 10 |
| outside | *rain* |  |  |  | 0 | 0 | 21 |
| outside | *road* |  |  |  | 0 | 0 | 11 |
| outside | *shop* |  |  |  | 0 | 0 | 19 |
| outside | *sky* |  |  |  | 0 | 0 | 28 |
| outside | *slide* |  |  |  | 0 | 0 | 20 |
| outside | *snow* | |  |  | 0 | 0 | 14 |
| outside | *star* |  |  |  | 0 | 1 | 31 |
| outside | *stone* |  |  |  | 0 | 0 | 15 |
| outside | *sun* |  |  |  | 0 | 0 | 23 |
| outside | *swing* |  |  |  | 0 | 1 | 22 |
| outside | *tree* |  |  |  | 0 | 3 | 31 |

S3. CDI words (continued)

|  |  |  |  |  | Number of infants acquired | | |
| --- | --- | --- | --- | --- | --- | --- | --- |
| Category | Word | Onom. | Dim. | Redup. | 9 mo. | 15 mo. | 21 mo. |
| outside | *wall* |  |  |  | 0 | 0 | 10 |
| outside | *water* |  |  |  | 0 | 2 | 31 |
| people | *aunt* |  |  |  | 0 | 0 | 5 |
| people | *auntie* |  | x |  | 0 | 0 | 12 |
| people | *baby* |  | x | x | 0 | 13 | 40 |
| people | *boy* |  |  |  | 0 | 1 | 22 |
| people | *girl* | |  |  | 0 | 0 | 20 |
| people | *lady* |  | x |  | 0 | 0 | 20 |
| people | *man* |  |  |  | 0 | 0 | 27 |
| people | *people* |  |  | x | 0 | 0 | 10 |
| people | *police* |  |  |  | 0 | 0 | 2 |
| people | *policeman* |  |  |  | 0 | 0 | 4 |
| people | *wee one* |  |  |  | 0 | 0 | 2 |
| toys | *ball* |  |  |  | 0 | 18 | 44 |
| toys | *balloon* |  |  |  | 0 | 3 | 31 |
| toys | *block* |  |  |  | 0 | 1 | 12 |
| toys | *book* |  |  |  | 0 | 11 | 39 |
| toys | *brick* |  |  |  | 0 | 1 | 14 |
| toys | *bubbles* |  |  | x | 0 | 13 | 35 |
| toys | *doll* |  |  |  | 0 | 2 | 11 |
| toys | *dolly* |  | x |  | 0 | 4 | 19 |
| toys | *pen* |  |  |  | 0 | 0 | 19 |
| toys | *teddy* |  | x |  | 0 | 7 | 30 |
| toys | *teddy bear* |  |  |  | 0 | 0 | 11 |
| toys | *toy* |  |  |  | 0 | 4 | 24 |
| vehicles | *aeroplane* |  |  |  | 0 | 0 | 15 |
| vehicles | *bicycle* |  |  |  | 0 | 0 | 8 |
| vehicles | *bike* |  |  |  | 0 | 0 | 26 |
| vehicles | *boat* |  |  |  | 0 | 1 | 30 |
| vehicles | *buggy* |  | x |  | 0 | 4 | 27 |
| vehicles | *bus* |  |  |  | 0 | 5 | 36 |
| vehicles | *car* |  |  |  | 0 | 8 | 41 |
| vehicles | *choochoo* | x |  | x | 0 | 3 | 34 |
| vehicles | *fire engine* |  |  |  | 0 | 0 | 12 |
| vehicles | *lorry* |  | x |  | 0 | 0 | 20 |
| vehicles | *plane* |  |  |  | 0 | 1 | 22 |
| vehicles | *pram* | |  |  | 0 | 0 | 11 |
| vehicles | *pushchair* |  |  |  | 0 | 0 | 5 |
| vehicles | *tractor* |  |  |  | 0 | 3 | 27 |
| vehicles | *train* |  |  |  | 0 | 1 | 27 |
| vehicles | *truck* |  |  |  | 0 | 1 | 20 |
| verbs | *bite* |  |  |  | 0 | 0 | 13 |

S3. CDI words (continued)

|  |  |  |  |  | Number of infants acquired | | |
| --- | --- | --- | --- | --- | --- | --- | --- |
| Category | Word | Onom. | Dim. | Redup. | 9 mo. | 15 mo. | 21 mo. |
| verbs | *blow* |  |  |  | 0 | 1 | 11 |
| verbs | *break* |  |  |  | 0 | 0 | 10 |
| verbs | *bring* |  |  |  | 0 | 0 | 7 |
| verbs | *bump* |  |  |  | 0 | 0 | 15 |
| verbs | *carry* |  | x |  | 0 | 1 | 20 |
| verbs | *catch* | |  |  | 0 | 0 | 13 |
| verbs | *clean* |  |  |  | 0 | 0 | 17 |
| verbs | *close* |  |  |  | 0 | 0 | 12 |
| verbs | *come* |  |  |  | 0 | 0 | 16 |
| verbs | *cry* |  |  |  | 0 | 0 | 13 |
| verbs | *cuddle* |  |  |  | 0 | 1 | 31 |
| verbs | *dance* |  |  |  | 0 | 0 | 18 |
| verbs | *draw* |  |  |  | 0 | 0 | 18 |
| verbs | *drink* |  |  |  | 0 | 1 | 19 |
| verbs | *drop* |  |  |  | 0 | 1 | 10 |
| verbs | *eat* |  |  |  | 0 | 0 | 16 |
| verbs | *fall* |  |  |  | 0 | 0 | 16 |
| verbs | *feed* |  |  |  | 0 | 0 | 6 |
| verbs | *find* |  |  |  | 0 | 0 | 9 |
| verbs | *finish* |  |  |  | 0 | 0 | 14 |
| verbs | *get* |  |  |  | 0 | 0 | 10 |
| verbs | *give* |  |  |  | 0 | 0 | 6 |
| verbs | *go* |  |  |  | 0 | 3 | 21 |
| verbs | *help* |  |  |  | 0 | 0 | 22 |
| verbs | *hit* |  |  |  | 0 | 0 | 8 |
| verbs | *hug* |  |  |  | 0 | 1 | 7 |
| verbs | *hurry* |  | x |  | 0 | 0 | 3 |
| verbs | *jump* |  |  |  | 0 | 0 | 22 |
| verbs | *kick* |  |  |  | 0 | 1 | 18 |
| verbs | *kiss* |  |  |  | 0 | 4 | 25 |
| verbs | *like* |  |  |  | 0 | 0 | 8 |
| verbs | *look* |  |  |  | 0 | 0 | 11 |
| verbs | *love* |  |  |  | 0 | 2 | 12 |
| verbs | *open* |  |  |  | 0 | 1 | 20 |
| verbs | *play* |  |  |  | 0 | 0 | 17 |
| verbs | *pull* | |  |  | 0 | 0 | 10 |
| verbs | *push* |  |  |  | 0 | 1 | 16 |
| verbs | *put* |  |  |  | 0 | 0 | 6 |
| verbs | *read* |  |  |  | 0 | 0 | 16 |
| verbs | *ride* |  |  |  | 0 | 0 | 5 |
| verbs | *run* |  |  |  | 0 | 0 | 19 |

S3. CDI words (continued)

|  |  |  |  |  | Number of infants acquired | | |
| --- | --- | --- | --- | --- | --- | --- | --- |
| Category | Word | Onom. | Dim. | Redup. | 9 mo. | 15 mo. | 21 mo. |
| verbs | *say* |  |  |  | 0 | 0 | 3 |
| verbs | *see* |  |  |  | 0 | 0 | 7 |
| verbs | *show* |  |  |  | 0 | 0 | 7 |
| verbs | *shut* |  |  |  | 0 | 0 | 16 |
| verbs | *sing* |  |  |  | 0 | 0 | 11 |
| verbs | *sleep* |  |  |  | 0 | 0 | 22 |
| verbs | *smile* | |  |  | 0 | 0 | 8 |
| verbs | *splash* |  |  |  | 0 | 2 | 29 |
| verbs | *stop* |  |  |  | 0 | 0 | 22 |
| verbs | *swim* |  |  |  | 0 | 0 | 17 |
| verbs | *swing* |  |  |  | 0 | 0 | 13 |
| verbs | *take* |  |  |  | 0 | 0 | 5 |
| verbs | *throw* |  |  |  | 0 | 0 | 11 |
| verbs | *tickle* |  |  |  | 0 | 2 | 21 |
| verbs | *touch* |  |  |  | 0 | 0 | 8 |
| verbs | *walk* |  |  |  | 0 | 1 | 27 |
| verbs | *wash* |  |  |  | 0 | 0 | 20 |
| verbs | *wipe* |  |  |  | 0 | 1 | 18 |
| adjectives | *all gone* |  |  |  | 0 | 5 | 34 |
| adjectives | *asleep* |  |  |  | 0 | 0 | 19 |
| adjectives | *big* |  |  |  | 0 | 0 | 14 |
| adjectives | *blue* |  |  |  | 0 | 1 | 21 |
| adjectives | *broken* |  |  |  | 0 | 0 | 19 |
| adjectives | *careful* |  |  |  | 0 | 0 | 11 |
| adjectives | *clean* |  |  |  | 0 | 0 | 16 |
| adjectives | *cold* |  |  |  | 0 | 1 | 26 |
| adjectives | *cross* |  |  |  | 0 | 0 | 2 |
| adjectives | *dirty* |  | x |  | 0 | 4 | 24 |
| adjectives | *empty* |  | x |  | 0 | 0 | 11 |
| adjectives | *fast* |  |  |  | 0 | 0 | 6 |
| adjectives | *gentle* |  |  |  | 0 | 3 | 12 |
| adjectives | *good* |  |  |  | 0 | 1 | 14 |
| adjectives | *happy* |  | x |  | 0 | 0 | 16 |
| adjectives | *hot* |  |  |  | 0 | 5 | 36 |
| adjectives | *hungry* |  | x |  | 0 | 1 | 9 |
| adjectives | *hurt* |  |  |  | 0 | 0 | 5 |
| adjectives | *mucky* | | x |  | 0 | 0 | 4 |
| adjectives | *naughty* |  | x |  | 0 | 0 | 14 |
| adjectives | *nice* |  |  |  | 0 | 2 | 15 |
| adjectives | *pretty* |  | x |  | 0 | 1 | 12 |
| adjectives | *sad* |  |  |  | 0 | 0 | 7 |
| adjectives | *silly* |  | x |  | 0 | 0 | 6 |

S3. CDI words (continued)

|  |  |  |  |  | Number of infants acquired | | |
| --- | --- | --- | --- | --- | --- | --- | --- |
| Category | Word | Onom. | Dim. | Redup. | 9 mo. | 15 mo. | 21 mo. |
| adjectives | *sleepy* |  | x | x | 0 | 0 | 13 |
| adjectives | *soft* |  |  |  | 0 | 0 | 8 |
| adjectives | *thirsty* |  | x |  | 0 | 0 | 4 |
| adjectives | *tired* |  |  |  | 0 | 0 | 10 |
| adjectives | *wee* |  |  |  | 0 | 0 | 5 |
| adjectives | *wet* |  |  |  | 0 | 0 | 17 |
| adjectives | *yellow* | |  |  | 0 | 0 | 14 |
| adjectives | *yucky* |  | x |  | 0 | 0 | 15 |
| prepositions | *back* |  |  |  | 0 | 0 | 13 |
| prepositions | *down* |  |  |  | 0 | 2 | 28 |
| prepositions | *in* |  |  |  | 0 | 0 | 12 |
| prepositions | *inside* |  |  |  | 0 | 0 | 9 |
| prepositions | *off* |  |  |  | 0 | 1 | 26 |
| prepositions | *on* |  |  |  | 0 | 0 | 22 |
| prepositions | *out* |  |  |  | 0 | 1 | 16 |
| prepositions | *there* |  |  |  | 0 | 1 | 12 |
| prepositions | *to* |  |  |  | 0 | 0 | 3 |
| prepositions | *under* |  |  |  | 0 | 0 | 7 |
| prepositions | *up* |  |  |  | 1 | 9 | 32 |
| pronouns | *he* |  |  |  | 0 | 0 | 2 |
| pronouns | *her* |  |  |  | 0 | 0 | 1 |
| pronouns | *his* |  |  |  | 0 | 0 | 1 |
| pronouns | *it* |  |  |  | 0 | 0 | 2 |
| pronouns | *me* |  |  |  | 0 | 2 | 18 |
| pronouns | *mine* |  |  |  | 0 | 3 | 27 |
| pronouns | *my* |  |  |  | 0 | 1 | 10 |
| pronouns | *she* |  |  |  | 0 | 0 | 1 |
| pronouns | *that* |  |  |  | 0 | 3 | 12 |
| pronouns | *this* |  |  |  | 0 | 1 | 9 |
| pronouns | *you* |  |  |  | 0 | 0 | 6 |
| pronouns | *your* |  |  |  | 0 | 0 | 2 |
| quantifiers | *again* |  |  |  | 0 | 5 | 30 |
| quantifiers | *all* |  |  |  | 0 | 0 | 13 |
| quantifiers | *another* |  |  |  | 0 | 0 | 5 |
| quantifiers | *more* |  |  |  | 1 | 10 | 37 |
| quantifiers | *none* |  |  |  | 0 | 0 | 5 |
| quantifiers | *not* | |  |  | 0 | 0 | 6 |
| quantifiers | *some* |  |  |  | 0 | 0 | 4 |
| question | *how* |  |  |  | 0 | 0 | 2 |
| question | *what* |  |  |  | 0 | 1 | 12 |
| question | *when* |  |  |  | 0 | 0 | 1 |
| question | *where* |  |  |  | 0 | 0 | 10 |

S3. CDI words (continued)

|  |  |  |  |  | Number of infants acquired | | |
| --- | --- | --- | --- | --- | --- | --- | --- |
| Category | Word | Onom. | Dim. | Redup. | 9 mo. | 15 mo. | 21 mo. |
| question | *who* |  |  |  | 0 | 0 | 6 |
| question | *why* |  |  |  | 0 | 0 | 2 |
| time | *day* |  |  |  | 0 | 0 | 5 |
| time | *later* |  |  |  | 0 | 0 | 5 |
| time | *morning* |  |  |  | 0 | 1 | 11 |
| time | *night* |  |  |  | 0 | 0 | 16 |
| time | *now* | |  |  | 0 | 0 | 5 |
| time | *today* |  |  |  | 0 | 0 | 1 |
